# Transplant Trial Watch

**DOI:** 10.3389/ti.2024.14105

**Published:** 2024-12-11

**Authors:** Simon R. Knight

**Affiliations:** ^1^ Centre for Evidence in Transplantation, Nuffield Department of Surgical Sciences, University of Oxford, Oxford, United Kingdom; ^2^ Oxford Transplant Centre, Churchill Hospital, Oxford, United Kingdom

**Keywords:** randomised controlled trial, kidney transplantation, liver transplantation, hypothermic oxygenated machine perfusion, health related quality of life

To keep the transplantation community informed about recently published level 1 evidence in organ transplantation ESOT and the Centre for Evidence in Transplantation have developed the Transplant Trial Watch. The Transplant Trial Watch is a monthly overview of 10 new randomised controlled trials (RCTs) and systematic reviews. This page of Transplant International offers commentaries on methodological issues and clinical implications on two articles of particular interest from the CET Transplant Trial Watch monthly selection. For all high quality evidence in solid organ transplantation, visit the Transplant Library: www.transplantlibrary.com.

**Randomised Controlled Trial 1**

Effect of an Exercise Intervention or Combined Exercise and Diet Intervention on Health-Related Quality of Life-Physical Functioning After Kidney Transplantation: The Active Care After Transplantation (ACT) Multicentre Randomised Controlled Trial.

*by Knobbe, T. J., et al. The Lancet Healthy Longevity 2024 [record in progress].*

## Aims

The aim of this study was to determine the role of exercise intervention or exercise plus diet intervention on the physical functioning domain of health-related quality of life following kidney transplantation.

## Interventions

Participants were randomised into three groups: usual care, exercise intervention, and exercise plus diet intervention.

## Participants

221 kidney transplant recipients.

## Outcomes

The primary endpoint was change in the physical functioning domain of health-related quality of life (HRQoL). The secondary endpoints were HRQoL composite scores, physical activity, physical fitness, cardiometabolic risk factors and body composition.

## Follow-Up

15 months.

## CET Conclusion

*by Simon Knight*

This multicentre RCT from the Netherlands randomised kidney transplant recipients to one of three groups: usual care, exercise or exercise plus diet. The exercise component comprised a 3-month supervised exercise program, with additional dietary counselling over 15 months in the exercise plus diet group. Interestingly for an RCT, the primary endpoint was a quality-of-life measure (the physical functioning component of the SF-36 questionnaire). At 3 months a small difference in physical functioning was seen in the exercise group, but this difference disappeared by 15 months. Study design and conduct are good, with variable block randomisation and allocation concealment via centralised randomisation. Interventions are well described, and primary analysis is by intent-to-treat. There are some limitations – 35% recruited patients were excluded from the intent-to-treat analysis due to missing primary outcome data at baseline or follow-up, a common issue in studies using QOL questionnaires. This may have led to a lack of statistical power. Also of note, the study only recruited patients in their first year post-transplant, so the results may not generalise to patients later post-transplant.

## Jadad Score

3.

## Data Analysis

Strict intention-to-treat analysis.

## Allocation Concealment

Yes.

## Trial Registration

ClinicalTrials.gov - NCT01047410.

**Randomised Controlled Trial 2**

Hypothermic Oxygenated Machine Perfusion Influences the Immunogenicity of Donor Livers In Humans.

*by Elgosbi, M., et al. Liver Transplantation 2024 [record in progress].*

## Funding Source

Non-industry funded.

## Aims

This observational study aimed to examine the influence of hypothermic oxygenated machine perfusion (HOPE) on the molecular profile of liver allografts as well as on the immune responses induced following liver transplantation.

## Interventions

Participants from two randomised controlled trials comparing donor livers randomly assigned to either HOPE or to static cold storage (SCS), were included.

## Participants

27 liver transplant recipients.

## Outcomes

Molecular and immunogenic profiles of donor livers.

## Follow-Up

3 months posttransplantation.

## CET Conclusion

*by Simon Knight*

This interesting study investigated the immune responses in 27 liver transplant recipients participating in two randomised controlled trials of hypothermic oxygenated machine perfusion (HOPE) in a single centre. The investigators studied perfusate, liver biopsies and recipient T-cell profiles. They showed that, compared to static cold storage, HOPE livers demonstrated reduction in hepatic immune cells in the perfusate and a reduced activation of the reactive oxygen species pathway. In the recipient, there was upregulation in donor-specific T-reg cell expression following HOPE. These findings are interesting, but as this represents only a small single-centre subset of the overall RCT recruitment, can only be exploratory. The patients included only a very small number of DCD liver recipients. They are, however, in keeping with the reduction in acute rejection rates seen in other studies of HOPE in the liver and kidney.

## Trial Registration

ClinicalTrials.gov - NCT01317342; ClinicalTrials.gov - NCT02584283.

## Funding Source

Non-industry funded.

## Clinical Impact Summary

*by Simon Knight*

*Ex-vivo* machine perfusion of the liver has a number of potential benefits, including reconditioning, viability assessment and extended preservation durations. Whilst hypothermic oxygenated machine preservation (HOPE) may not afford the same extended preservation times or viability assessment as normothermic perfusion, it has shown the potential to reduce incidence of early allograft dysfunction and surgical complications, including the risk of non-anastomotic biliary strictures [1].

One area that is less studied is the immunological impact of machine perfusion. By increasing ATP storage and reducing ischaemia-reperfusion injury, it is possible that machine preservation has the potential to reduce the innate and adaptive immune response following reperfusion. This has been demonstrated in rodent liver transplant models, where lower doses of immunosuppression are required for successful transplantation following HOPE [2]. There is also some clinical evidence for this following hypothermic oxygenated machine preservation of the kidney, with the COMPARE study demonstrating a reduction in risk of acute rejection for HOPE compared to conventional hypothermic machine preservation [3].

In a recent, posthoc analysis of samples from 2 randomised clinical trials, Elgosbi et al. investigated the role of HOPE in the immunogenicity of liver transplantation [4]. HOPE resulted in lower presence of intrahepatic immune cells (liver mononuclear cells), compared to static cold storage (SCS). Transcriptomic analysis demonstrated less activation of elements of the reactive oxygen species pathway, which translated to a later increase in expression of CD4+FOXP3+ regulatory T-cells and a reduction in alloreactive CD8^+^ T cells.

The sample size in the study is too small to determine whether these immunological effects translate into a clinically meaningful difference in rejection rates or graft function, but nevertheless provide an interesting insight into the mechanisms behind reduced immune activation seen with oxygenated machine perfusion. The majority of patients in the cohort received DBD liver transplants, so it would be interesting to see if the benefit is greater in more injured DCD grafts.

Overall, this is an interesting study, and hopefully paves the way for more detailed analysis alongside future clinical trials of machine perfusion.

### Clinical Impact

3/5.
